# Supplementary material for: Prior uncertainty impedes discrete locomotor adaptation
Source: PLoS One. 2024 Feb 16;19(2):e0291284. doi: 10.1371/journal.pone.0291284 (PMC10871477; doi:10.1371/journal.pone.0291284)
Supplement: S1 File — (DOCX) [file pone.0291284.s001.docx]

**3. Results**

**3.1 Unpredictable Forces**

Full results assessing the unpredictable forces are reported in the main text.

**3.2 Adaptation to Force Field**

**3.2a Center of Mass Trajectories**

Mixed ANOVA confirmed a main effect of experimental Period on COM signed deviation, *F*(1.90, 47.74) = 68.21, *p* < .001, *η*­_p_^2^ = 0.75. COM signed deviation was significantly larger during the Early Field (-0.07 ± 0.03 m^2^) than baseline, *t*(24) = 10.96, *p* < .001, *d* = 2.80, confirming that participants’ COM trajectories overall were substantially disrupted during initial exposure to the Force Field. COM signed deviation decreased (in rightward bias) from Early Field to Late Field (-0.01 ± 0.03 m^2^), *t*(24) = 8.49, *p* < .001, *d* = 2.17, confirming that participants overall adapted as they practiced walking in the Force Field. Late Field was still different from baseline, *t*(24) = 2.47, *p* = .032, *d* = .75, indicating that participants overall did not fully return to performance similar to baseline levels by the end of the Force Field trials (however, see the interaction effect discussed below, which expands on this). Washout trials (0.01 ± 0.01 m^2^) were different from Early Field trials, *t*(24) = 12.23, *p* < .001, *d* = 3.12, and Late Field trials, *t*(24) = 3.74, *p* < .001, *d* = 0.95, indicating less rightward bias (and greater leftward bias) than both, but were not different from baseline trials, *p* = .21. This confirms that walking overall returned to baseline levels by the end of the washout period.

The ANOVA also indicated a main effect of Group, *F*(1,23) = 18.70, *p* < .001, *η*­_p_^2^ = 0.45. The Perturbation group exhibited greater rightward bias in COM signed deviation (-0.03 ± 0.04 m^2^) overall compared to the Non-Perturbation group (-.01 ± 0.03 m^2^), confirming what was visually apparent in the data.

Finally, there was a significant interaction between Period and Group. The full report of this effect can be found in the main text.

**3.2b Center of Mass Lateral Offset**

A mixed ANOVA confirmed a main effect of experimental period on lateral offset, *F*(2.02,46.38) = 16.17, *p* < .001, *η*­_p_^2^ = 0.41. Ignoring group, participants overall exhibited no change in lateral offset from baseline (0.02 ± 0.01 m) to Early Field (0.02 ± 0.01 m), *p* = .35. However, lateral offset during Late Field trials (0.03 ± 0.01 m) was greater than both baseline, *t*(24) = 5.84, *p* < .001, *d* = 1.24, and Early Field, *t*(24) = 4.25, *p* < .001, *d* = 0.90 (however, see the interaction discussed below, which expands on this). Lateral offset during washout trials (0.02 ± 0.01 m) was no different than baseline trials or Early Field trials, both *p* > .6, but was likewise less than Late Field trials, *t*(24) = 5.16, *p* < .001, *d* = 1.10.

There was no main effect of group on lateral offset, *p* = .23.

The ANOVA also indicated a significant interaction between experimental Period and Group on lateral offset. This effect is fully reported in the main text.

**3.2c First Step Width**

A mixed ANOVA confirmed a main effect of experimental Period on first step width, *F*(1.74,40.00) = 81.65, *p* < .001, *η*­_p_^2^ = .78. Ignoring group, participants overall showed a significant increase in first step width from baseline to Early Field (0.09 ± 0.06 m), *t*(24) = 8.05, *p* < .001, *d* = 2.01, as well as from baseline to Late Field (0.13 ± 0.06 m), *t*(24) = 11.23, *p* < .001, *d* = 2.80. First step width also increased from Early Field to Late Field, *t*(24) = 3.18, *p* = .004, *d* = 0.79. First step width in the washout trials (0.01 ± 0.03 m) was less than both Late Field, *t*(24) = 10.76, *p* < .001, *d* = 2.68, and Early Field, *t*(24) = 7.57, *p* < .001, *d* = 1.89. There was no difference between washout and baseline, *p* = .64, indicating that participants overall returned first step width back to baseline-similar levels by the end of the washout trials.

There was no main effect of group on first step width, *p* = .14.

The mixed ANOVA also indicated a significant interaction between Period and Group, and this effect is fully reported in the main text.

**3.3 Catch Trials**

**3.3a Center of Mass Trajectories**

All effects are fully reported in the main text.

**3.3b COM Lateral Offset**

All effects are fully reported in the main text.

**3.3c First Step Width**

A mixed ANOVA indicated a main effect of Trial on first step width, *F*(1,23)=135.42, *p* < .001, *η*­_p_^2^ = 0.86. First step width overall decreased from Pre-Catch trials (0.12 ± 0.05 m) to Catch trials (-0.01 ± 0.10 m), frequently to the point of becoming narrower than baseline values (i.e., negative values). The ANOVA also indicated a main effect of group, *F*(1,23) = 8.92, *p* = .007, *η*­_p_^2^ = 0.28, where the Perturbation group (0.09 ± 0.04 m) showed greater first step width across Pre-Catch and Catch trials than the Non-Perturbation group (0.02 ± 0.13 m).

These main effects were qualified by a significant interaction between Trial and Group, which is fully reported in the main text.
